# Topographical shifts in fine root lifespan in a mixed, mesic temperate forest

**DOI:** 10.1371/journal.pone.0254672

**Published:** 2021-07-14

**Authors:** Edward J. Primka, Thomas S. Adams, Alexandra Buck, David M. Eissenstat

**Affiliations:** 1 Department of Ecosystem Science and Management, The Pennsylvania State University, University Park, Pennsylvania, United States of America; 2 Graduate Program in Ecology, The Pennsylvania State University, University Park, Pennsylvania, United States of America; 3 Department of Plant Science, The Pennsylvania State University, University Park, Pennsylvania, United States of America; Chinese Academy of Forestry, CHINA

## Abstract

Root lifespan, often is estimated in landscape- and ecosystem-level carbon models using linear approximations. In water manipulation experiments, fine root lifespan can vary with soil water content. Soil water content is generally structured by complex topography, which is largely unaccounted for in landscape- and ecosystem-scale carbon models. Topography governs the range of soil water content experienced by roots which may impact their lifespan. We hypothesized that root lifespan varied nonlinearly across a temperate, mesic, forested catchment due to differences in soil water content associated with topographic position. We expected regions of the landscape that were too wet or too dry would have soils that were not optimal for roots and thus result in shorter root lifespans. Specifically, we hypothesized that root lifespan would be longest in areas that consistently had soil water content in the middle of the soil water content spectrum, while in soils at either very low or very high soil water content, root lifespan would be relatively short. We tested this hypothesis by collecting and analyzing two years of minirhizotron and soil moisture data in plots widely distributed in the Shale Hills catchment of the Susquehanna-Shale Hills Critical Zone Observatory in Pennsylvania. We found that fine root lifespans were longer in traditionally wetter topographic regions, but detected no short term (biweekly) effect of soil moisture on root lifespan. Additionally, depth in soil, soil series, slope face orientation, and season of birth strongly affected root lifespans across the catchment. In contrast, lifespan was unaffected by root diameter or mycorrhizal association. Failure to account for these variables could result in erroneous estimates of fine root lifespan and, consequentially, carbon flux in temperate forested regions.

## Introduction

Fine roots play a large role in the carbon (C) cycling of terrestrial ecosystems. For example, fine roots have been estimated to be ~22% of the global average, annual net primary productivity [1]. However, there is a large knowledge gap as to how abiotic conditions impact the amount of C flux that fine roots contribute to the C cycling process. An important first step to elucidating the role of fine roots in the C cycle is to have better estimates of fine root longevity and to identify key drivers affecting root lifespan in temperate forests.

One way fine roots can influence terrestrial C cycling is by their respiration which is tied to the age of the root. Root respiration, depending upon estimates, differing methods, model system or the tree species measured, contributes 14%-77% of total soil C efflux [2–12]. Total soil C efflux makes up around 60–88% of total terrestrial forest ecosystem respiration [13–16]. Fine root lifespan governs the length of time that fine roots respire in the soil and the rate of root respiration. Simply put, a root must respire to live, so the longer a root is alive, the more carbon dioxide it will respire into the soil. Root age also strongly influences root respiration. Younger roots tend to respire at a rate much faster than older roots [17]. In areas with high root production, high turnover, and therefore short root lifespan, the root population may exhibit higher soil carbon efflux because the general population of roots would tend to be younger.

Soil water is one abiotic factor that is hypothesized to strongly influence both root production and root lifespan. Precipitation is expected to become much more variable with climate change [18], causing an increased need to better understand how plants respond belowground to changes in soil water. In addition, hillslope topography can accentuate shifts in rainfall because of differences in hillslope length and steepness, curvature, and aspect. Thus, complex topography within landscapes spatially structures soil moisture through time [19–21]. Hillslopes and upper slope areas with relatively limited soil depth typically have drier soils [20,21]. Valley floor regions and some concave mid-slope (swale) areas usually have deeper soils and are generally wetter through time [20–22]. Additionally, slope in combination with soil type impacts soil moisture content so that flatter areas will tend to be wetter than steeper areas that share the same soil type classification [23]. Soil series itself impacts soil moisture as some soil series tend to remain wetter longer than other soil series even when these soils are separated by a relatively small distance [20]. Yet there are no studies to our knowledge that have examined how spatially structured, long-term differences in soil water content in complex topography impact fine root lifespans.

Long-term effects of soil moisture on fine root lifespan are poorly understood. Short-term soil moisture variability, on the other hand, has non-linear impacts on fine root lifespan. Fine roots often die faster in areas with excessively high soil water content or “waterlogged” soils [24,25] and in areas with very low soil water content (soil water content ranged from 31–44% in Glenz et al. and soil available water from 1–17% V/V in Huang et al.) [26,27]. Additionally, roots have been shown over short periods of time to live longer in soil patches that are wetter and have more nitrogen (N) [28], suggesting that localized wetter soil can support longer fine root lifespans.

In addition to soil moisture, soil depth and temperature can affect root lifespan. Deeper roots usually live longer than roots in shallow soil layers [29–32]. Deeper soils tend to have a more consistent soil temperature and less microbial activity, while upper soil layers exhibit greater diurnal fluctuation in temperatures and greater microbial activity [33,34]. Typically, as soils warm during the growing season there is increased fine root mortality [26]. Reduced temperature fluctuation, more consistent soil moisture, and lower biological activity (e.g., fewer herbivores and pathogens) may contribute to longer root lifespans in deeper soils.

Seasonal temperature fluctuations also can affect root lifespan. Root lifespan tends to vary based on when fine roots are produced, with roots born earlier in the year tending to live longer in temperate climates than those born later [35,36]. Temperature variation can also be affected by aspect of a hillslope. South-facing hillslopes typically experience more radiation than north facing hillslopes in the northern hemisphere [37,38]. This results in south-facing hill slopes warming faster than north-facing hillslopes, especially in spring with accelerated snow melt, which may lead to shorter root lifespan on south- than north-facing hillslopes.

The main objective of this study was to examine patterns of root lifespan across complex topography. We hypothesized that: fine root lifespan is non-linearly related to soil water content, such that root lifespans are shorter in areas with very wet or dry soils compared to areas with soils in the mid-moisture range. Thus, in topographical positions with high moisture such as valley floors and swales, we expected shorter root lifespan when soil water content was very high. Similarly, we anticipated in topographical positions with low moisture such as midslopes and ridge tops, root lifespan would be shorter when soil water content was very low. In addition, we expected that south-facing slopes and warmer seasons would result in shorter root lifespans due to increased soil temperature.

## Materials and methods

### Watershed

This study was conducted at Susquehanna-Shale Hills Critical Zone Observatory (SSHCZO), which is located in Central Pennsylvania, USA (40°40’N 77°54’W). Work was done on The Pennsylvania State University land and we received permission from the Director of Stone Valley Forests, Joe Harding. Elevation ranges from 256 to 310 m around the catchment [20]. Soil series across the SSHCZO are Weikert (loamy-skeletal, mesic Lithic Dystrudept), Rushtown (loamy-skeletal, mesic Typic Dystrochrept), Berks (loamy-skeletal, mesic Typic Dystrudept), Blairton (fine-loamy, mesic Aquic Hapludult), and Ernest (fine-loamy, Aquic Fragiudults) [23]. Weikert soils make up the majority of the midslope planar locations and all of the ridgetop locations [20]. At the Weikert midslope locations, slope can vary widely, unlike the other soil series in the catchment [23]. Ernest and Blairton soils are in the valley floor [20]. Swales, or concave regions present in the midslope region, are mostly Rushtown soils [20]. Select regions of the midslope planar areas and areas bordering the Rushtown and Ernest soils are the Berks soil series [20]. The SSHCZO forest is composed of mostly deciduous trees (several species of maple (*Acer*), hickory (*Carya*), and oaks (*Quercus*)) with some evergreen species (*Tsuga canadensis*, *Pinus strobus*, and *Pinus virginiana*) [39]. Mean annual temperature and precipitation for this area have been around 11°C and 900 mm in the past [39]. Precipitation in 2017 and 2018 from March 1^st^ to November 30^th^ were 1012.5 and 1319.4 mm, respectively.

### Macroplot sites

**Fig 1:**
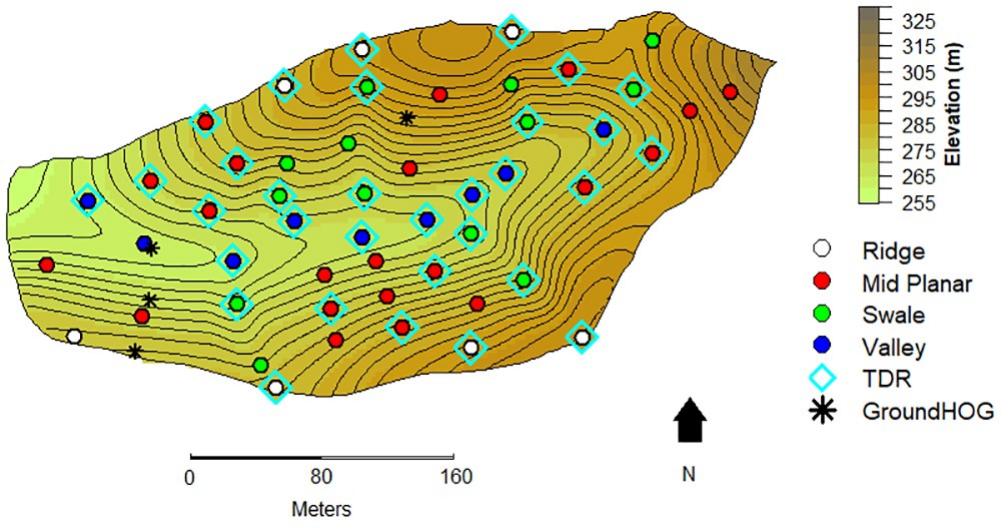
Susquehanna Shale Hills Critical Zone Observatory macroplot site map. Elevation map of Susquehanna Shale Hills Critical Zone Observatory with included topographical contoured lines. White, red, green, and blue dots represent macroplot sites that were established at ridge top, mid-slope planar, swale, and valley floor locations, respectively. Cyan diamonds represent macroplot volumetric time domain reflectometry (TDR) sensors locations, 2–3 per plot. TDR observations were also taken at sites with previously installed soil moisture sensors termed “GroundHOG” sites, which were represented on the map as asterisks. GroundHOG stands for ground hydrologic observation gear. GroundHOG sites have additional gas, water and measuring sensors used by the SSHCZO that were not used in this study.

Across the SSHCZO, 50 circular macroplot sites (often referred to as simply “sites”), 5 m in diameter, were used to assess the influence of topography on root lifespan (Fig 1). Macroplot sites were established in all topographic regions with more coverage in the midslope region to account for more potential variability within this topographic region. Additionally, midslope planar region occupied more area in the catchment relative to all of the other topographic regions. Valley floor, swale, ridge top, and midslope planar regions had 9, 13, 7, and 21 sites respectively. Two hundred fifty clear acrylic minirhizotron tubes were installed in 2015 across the 50 macroplot sites within the SSHCZO. Five tubes were installed per macroplot site in the formation of a cross. The formation was five meters wide and ten meters long. Two tubes were installed at the ends of the horizontal line and three along the vertical. Tubes were installed at 30° from the absolute vertical. Installation depth of the tubes was 1.25 meters or to the point of refusal because of excessive rock fragments. The end of the tube that projected out of the soil was taped and painted white to reflect sunlight. Additionally, foam insulation was inserted into tubes to minimize shifts in temperature from the soil at that depth. A hole was drilled on one side of the tube to ensure that the same soil face was measured every time images were taken.

### Data collection

#### Root observations

Minirhizotron images were taken using a Bartz digital camera with ICAP version 7.0 [Bartz Technology Corp., Carpinteria, CA, USA]. An initial survey of image quality of minirhizotron tubes and qualitative root count was taken in late spring 2016. Based on this survey, one hundred tubes were selected based on root presence and image quality. These 100 tubes were observed on a biweekly basis to a depth of 40 cm. Because roots typically have shorter lifespans near the soil surface and measuring tubes to full depth more than triples the observation and processing time, we observed surface roots more frequently than deeper roots. Tubes were observed to full depth every other observation period from 2016 to the beginning of the growing season of 2018, otherwise tubes were observed to 40 cm. During full depth survey campaigns, an additional 50 tubes selected from the initial survey were followed beginning in 2016 for a total of 150 tubes followed monthly. From 2018 through two observation times at the beginning of the growing season 2019, tubes were sampled to a depth of 40 cm during each observation session. First-order roots were then followed through time via tracing in Rootfly version 2.0.2 [Wells and Birchfield, Clemson University, SC, USA]. Only first-order roots were traced. First order roots were based on root branching in images. Roots discovered to be not first order roots later in the image set were removed from the dataset. Roots larger than 2 mm and smaller than 0.2 mm (typically grass and other herbaceous roots) in diameter were excluded. Root diameter, length, color, and when a root disappeared were recorded. Root disappearance or visible degradation was used as the date of root death. Number of neighbors was calculated for each time point based on the number of other individuals within the same observation window. Tubes with any length of their acrylic surface that became exposed to the surface (allowing light penetration) were removed from the dataset, because it impacted the lifespan models. Root birth was assumed to be halfway between the image where they first appeared and the image where they were not yet visible. Similarly, the time of mortality was assumed to be the halfway point between when the root was present and when the root was gone from the minirhizotron image. Roots born in 2019 were removed. Roots that did not have a death point were kept (right censored in survivorship analysis).

#### Volumetric soil water content

In 2016, time domain reflectometry (TDR) sensors were generally measured on a biweekly basis to track soil water content at GroundHOG locations (Fig 1). TDR waveguides at GroundHOG locations were positioned at 20, 40, 60, and 100 cm depth in the valley floor. Midslope planar locations only had sensors measured at 20, 40, and 60 cm. Ridge top locations only had sensors measured at 20 and 40 cm. Depth of TDR sensors at GroundHOG sites was determined by soil depth. In July of 2017, a total of 88, 20-cm TDR probes were installed in 33 macroplot locations. Installation locations were chosen so that they covered all topographic locations and soil series, determined based on [20]. Probes were installed horizontally at 20, 40, and 60 cm depths where possible. Measurements from the sensors were taken on a biweekly basis. Soil water content was relativized by dividing each observation by the mean of the highest three readings over the year for an individual sensor and multiplied by the average of the top three measurements per topographic location to create a scaled measurement. This was done to remove inherent variation in individual TDR sensor readings due to rock presence around the sensors and inherent sensor idiosyncrasy.

#### Leaf litter collection

Leaf litter content was collected from litter traps that were established at all 50 macroplots. Two litter traps were sampled per site for a total of 100 traps. The traps were suspended at 0.5 m in height and had a collection area of 0.1472 m^2^. Leaf litter collected was only leaf blades, petioles, and needles. No woody mass, nuts, or acorns were included. Litter was collected weekly in 2016–2018. Litter collection began in late September in 2016 and 2017. Litter collection began in late October in 2018. Litter was dried in a drying oven at 60°C for 48 hours, and then weighed.

#### Soil nutrient collection

From June through October 2014, 250 soil cores that were 5 cm in diameter, were taken at a 60° angle from the soil surface to 165 cm depth or point of refusal with a gas-powered coring auger [Rhino GPD-40, Giddings Machine Company, Windsor, CO]. Cores were taken in the exact location that minrhizotron tubes were inserted into the soil. The O_a_ horizon was removed from each core. Cores were divided into 0–20 cm, 20–40 cm, 40–80 cm, 80–120 cm, and 120–165 cm. All soil samples were air dried and then sieved using a 2mm. Soil organic matter content (SOM) was determined by oven drying one-gram soil samples at 105°C for 24 hours, then reweighing soil samples, which was then followed by measuring the amount of loss on ignition at 450°C [40]. Inorganic nitrogen extraction was carried out following the procedure of [41]. Briefly, 5 ± 0.2g soil samples were shaken with 50ml of 2M KCl for one hour to extract inorganic nitrogen. Samples were then allowed to settle overnight in a refrigerator followed by filtering through Whatman #1 filters. Finally, Vanadium(III) Chloride reagent and Citrate, Salicylate-nitroprusside and Hypochlorite reagents were added to the filtered, room temperature samples for spectrophotometric determination of nitrate and ammonium concentrations respectively in parts per million [41].

### Statistical analysis

#### Data organization

Root observations were aligned with the closest water content readings. Season of birth was split into spring (March or April—May), summer (June—July), and fall (August—November or December). Season of birth was split in order to have linear effects within each rank.

#### Models

Cox proportional hazards models or Cox models [42] were used to determine the significance and importance of a given variable on fine root lifespan (risk of mortality). Cox models were all run with a site cluster variable that accounted for roots within a macroplot site of five tubes being more similar to each other than to those at other sites. The cluster variable groups the observations and allows the model to do robust variance, by removing the confounding factor of within macroplot site similarities due to distance of tubes from each other. To treat season of birth as a second stratifying variable, the data set was split by season of birth as well as year. Year and season of birth were examined first to determine if they had any effect on fine root lifespan.

#### Testing for confounding factors

Root dynamics in 2016 did not tend to follow the same trends of 2017 and 2018. Because roots born in 2016 were likely roots that were still under root disturbance [43], here we only present data from 2017 and 2018. Additionally, to account for potential collinearity between depth and topography, separate analyses were run initially for shallow roots (0–25 cm) versus deep roots (26–97 cm). Further, the maximum depth of the tube to refusal was included as a variable to ascertain whether soil volume in the area played a role. Tube maximum depth was not significant across the six seasons and was subsequently removed from final models. The separate data sets yielded similar results and so were combined for final analyses. To ensure no strong, topographic region based tree species effects were biasing the analyses, we tested the combination of fine root diameter and mycorrhizal association for tree species effects on fine root lifespan using a neighborhood effect approximation. We used a tree species map of the catchment and known AM/EM associations with tree species plus diameter to assign species to different groups [32,44–47]. Specific groups we used were: thin root-AM or–EM, medium root-AM or–EM, and thick root-AM. There were no tree species that fell within the thick root–EM category. The neighborhood effect approximation used was $tree\;basal\;area÷\sqrt{(distance\;}to\;tube)$ based on the model proposed by [48].

#### Final models

Variables included in the final model were based on whether the variable was significant in at least four of the six seasons across the two years. The final model accounting for the most variation in root survivorship consisted of soil depth where the root was observed and either hillslope position (ridge, planar midslope, swale, or valley floor) or soil series, with an included site cluster variable. Season of birth final models included a season of birth variable in addition to the depth and topography variables. When we examined the relationship between soils for the midslope planar region, the final model had depth, soil type, and north versus south slope as variables. Topography was removed as a variable, because there was only one soil type that corresponded with swales and several that corresponded with midslope planar locations, which rendered the inclusion of topography superfluous. Interactions were initially included and their significance was tested via partial likelihood ratio tests [49]. Cox proportional hazards models were run in R using the survival package [50,51]. The data were run in the survival package as being right censored to account for roots that did not die over the course of the season. Survivorship curves were produced using the survfit() function in the R package survival [50,51]. Survivorship curves are generated based on risk ratios instead of lifespan [52]. Risk ratios refer to the risk of mortality for a root. Risk ratios larger than 1 indicate increased risk of mortality. Risk ratios less than 1 indicate a decreased risk of mortality. All statistical analyses were conducted via the program R 3.6.3 [R Core team 2020].

#### Bayesian regression kriging

Bayesian kriging with an external trend was adapted from the methodology outlined by [39]. We summarize here briefly. We constructed a grid for Shale Hills with points every 2 meters and assigned different grid points values for soil series, topography, and north versus south slope. We utilized the *krige*.*bayes* function from the geoR package version 1.8–1 [53]. Variables found significant during Cox proportional hazards model testing were used as variables for an external trend model, which is where our model differed from Bennie et al. [39]. We used diffuse, flat uniform priors for all priors in the model. For each season within 2017 and 2018, we mapped the posterior mean values that were calculated from the Bayesian kriging process. Data used in the kriging process were only from sites that had ten or more root observations during the kriging period. For more on this method, see [39] and [54].

## Results

There was substantial variation in soil nutrients, litter content, and root variables among sites. There was a wide range in values of nitrate, ammonium, soil organic matter, leaf litter, tube depth, unscaled volumetric water content and root parameters across the catchment (S1 Table in S1 File). Year 2017 (Risk = 1.88, p = <0.001), 2018 (Risk = 5.26, p = <0.001), and season of birth (Risk = 1.004, p = <0.001) were found to have significant effects on fine root lifespan. Utilizing cut point testing and smoothed added variable plotting, as suggested by [49], season of birth, depth, number of root neighbors and leaf litter content were not found to exhibit linear trends. The interaction between depth and topographic location was generally found to be an important variable based on partial likelihood ratio tests; however, when examining the significance of the variables within the model, despite the partial likelihood ratio tests, the interaction terms tended to be not significant (S2 Table in S1 File), so the interaction term was not included in the final model. When looking at midslope sites specifically, slope aspect was also included in the final model. In addition, we did not observe significant effects of either tube maximum depth or the combination of mycorrhizal association and root diameter associated with different species.

### Topography

**Fig 2:**
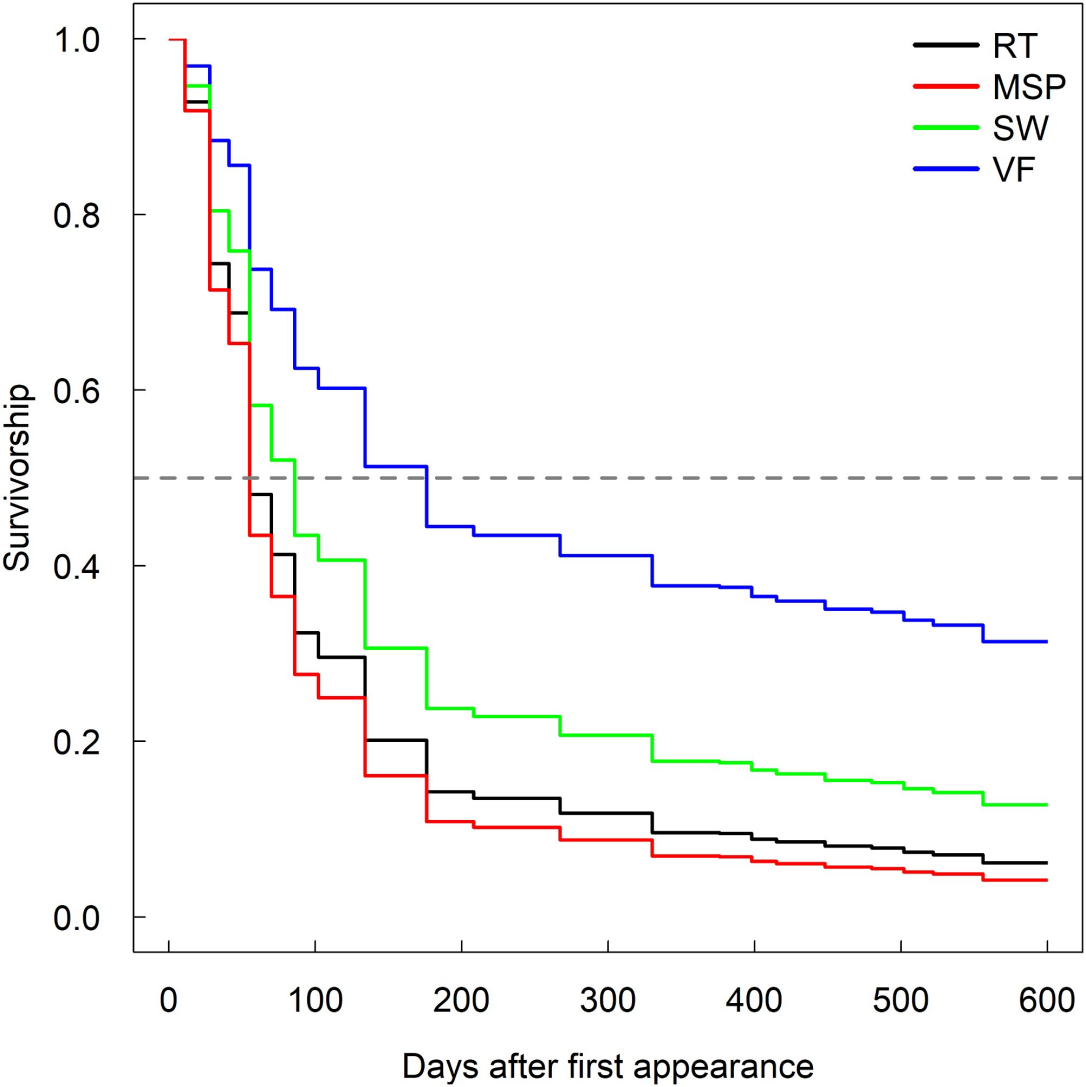
Survivorship of roots at different topographic locations. Survivorship curve of first-order roots in the top 10 cm of soil born in the spring of 2017 at different topographic regions, specifically ridge top (RT), midslope planar (MSP), swale (SW), and valley floor (VF) locations.

Wet topographical regions generally had longer root lifespans than drier regions. Generally, valley floor and swale locations had longer root lifespans and reduced risk of mortality than other topographic locations (Table 1 and Fig 2). As an example, median root lifespan was approximately 125 days longer in the valley floor and 45 days longer in the swales than that of planar midslope and ridgetop positions in the spring of 2017. Ridgetop sites were generally not significantly different than the midslope planar sites during the different years and seasons. When ridgetop sites were significantly different from the midslope planar sites, the risk ratios (0.50 and 1.87) did not show a consistent directional impact (i.e., sometimes roots lived longer on the ridge, other times shorter).

**Table 1:** Topographic proportional hazards table for different years and seasons.

Year	Season	Cohort (*n)*	Variable	Risk Ratio	Robust SE	P-value
2017	Spring	81	Ridgetop	0.88	0.18	0.48
2017	Spring	**218**	**Swale**	**0.65**	**0.21**	**0.04**
2017	Spring	**315**	**Valley Floor**	**0.37**	**0.21**	**<0.01**
2017	Summer	220	Ridgetop	0.91	0.18	0.60
2017	Summer	**519**	**Swale**	**0.66**	**0.17**	**0.01**
2017	Summer	**236**	**Valley Floor**	**0.47**	**0.12**	**<0.01**
2017	Fall	119	Ridgetop	0.86	0.34	0.66
2017	Fall	328	Swale	0.63	0.26	0.07
2017	Fall	281	Valley Floor	0.74	0.19	0.12
2018	Spring	**74**	**Ridgetop**	**1.87**	**0.22**	**<0.01**
2018	Spring	186	Swale	1.03	0.19	0.86
2018	Spring	**85**	**Valley Floor**	**0.45**	**0.34**	**0.02**
2018	Summer	**314**	**Ridgetop**	**0.50**	**0.15**	**<0.01**
2018	Summer	**410**	**Swale**	**0.66**	**0.18**	**0.02**
2018	Summer	**201**	**Valley Floor**	**0.64**	**0.21**	**0.03**
2018	Fall	186	Ridgetop	0.53	0.39	0.11
2018	Fall	429	Swale	1.07	0.26	0.81
2018	Fall	219	Valley Floor	0.69	0.25	0.14

Risk ratios for roots present at the different topographic regions relative to midslope planar locations for roots born in spring, summer, and fall seasons in 2017 and 2018. Risk ratios are for the full depth of the tubes, but depth is accounted for within the model. Risk ratios > 1 shows that risk of mortality was increased at the location, suggesting that lifespan was shorter. Risk ratios < 1 shows that risk was decreased at the location, suggesting that lifespans were generally longer. Bolded lines were statistically significant (*P<*0.05). Cohort sizes for midslope planar locations in spring (256), summer (669) and fall (509) for 2017 and 136, 629, and 608 for 2018, respectively.

### Depth

**Fig 3:**
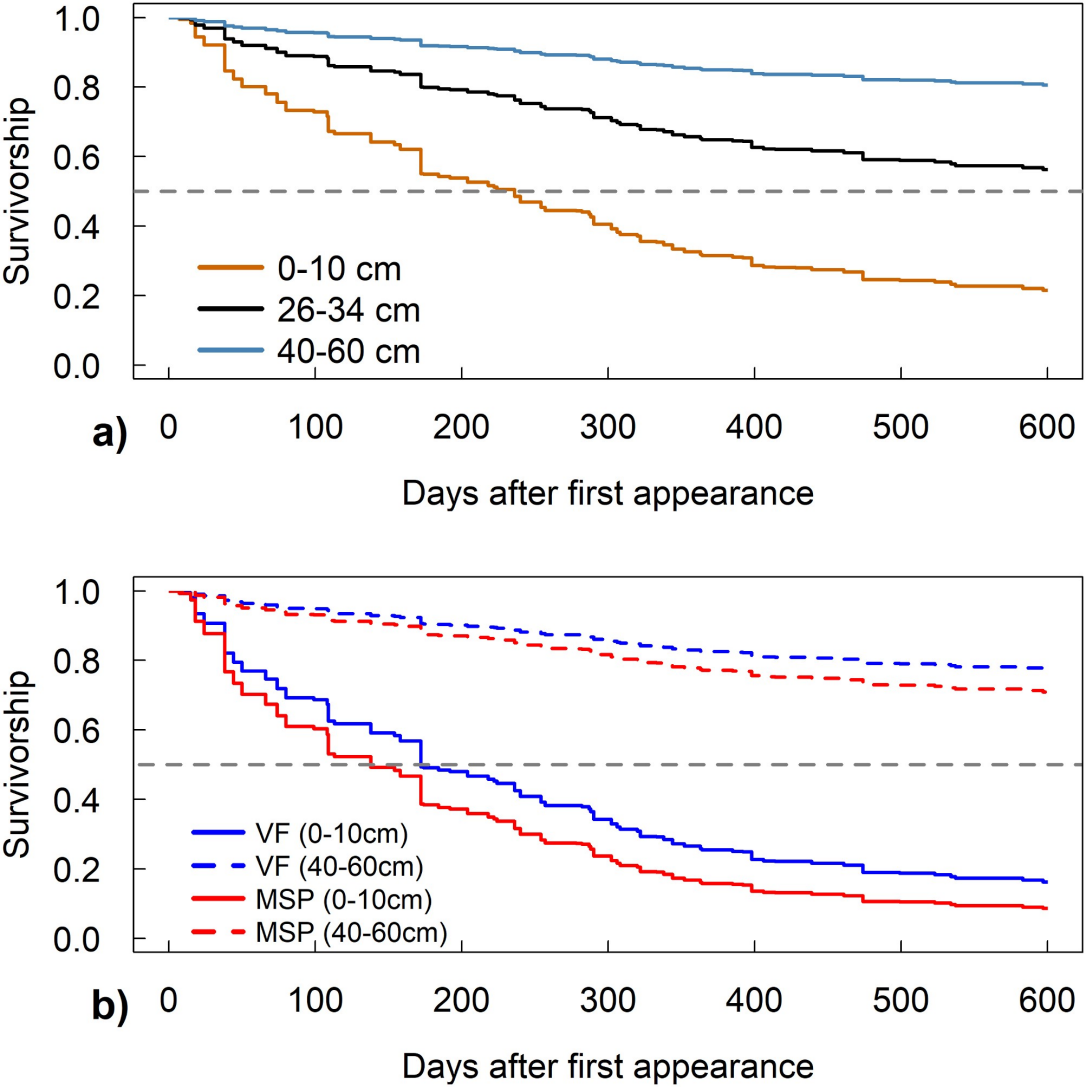
Survivorship of roots at different depths and topographic locations. (a) Survivorship curves of first-order roots at different soil depths born in the fall of 2017 at swale locations. (b) Survivorship curves of first-order roots at different soil depths at valley floor (VF) and midslope planar (MSP) locations.

Increasing depth resulted in increased fine root lifespan across all seasons and years of this study (S3 Table in S1 File and Fig 3a). For example, roots at a 40 cm depth lived approximately 180 days longer than those at a 10 cm depth. There were generally no consistent interactive effects on lifespan from topography and depth (Fig 3b). Thus, we did not observe greater risk of mortality in the deeper, valley floor roots born in the spring, despite the saturated soils at this time of year at these sites. For the few periods where different topographic locations showed significant interactive effects with depth, patterns were inconsistent (S2 Table in S1 File).

### Soil series, slope face, and season

#### Soil series

**Fig 4:**
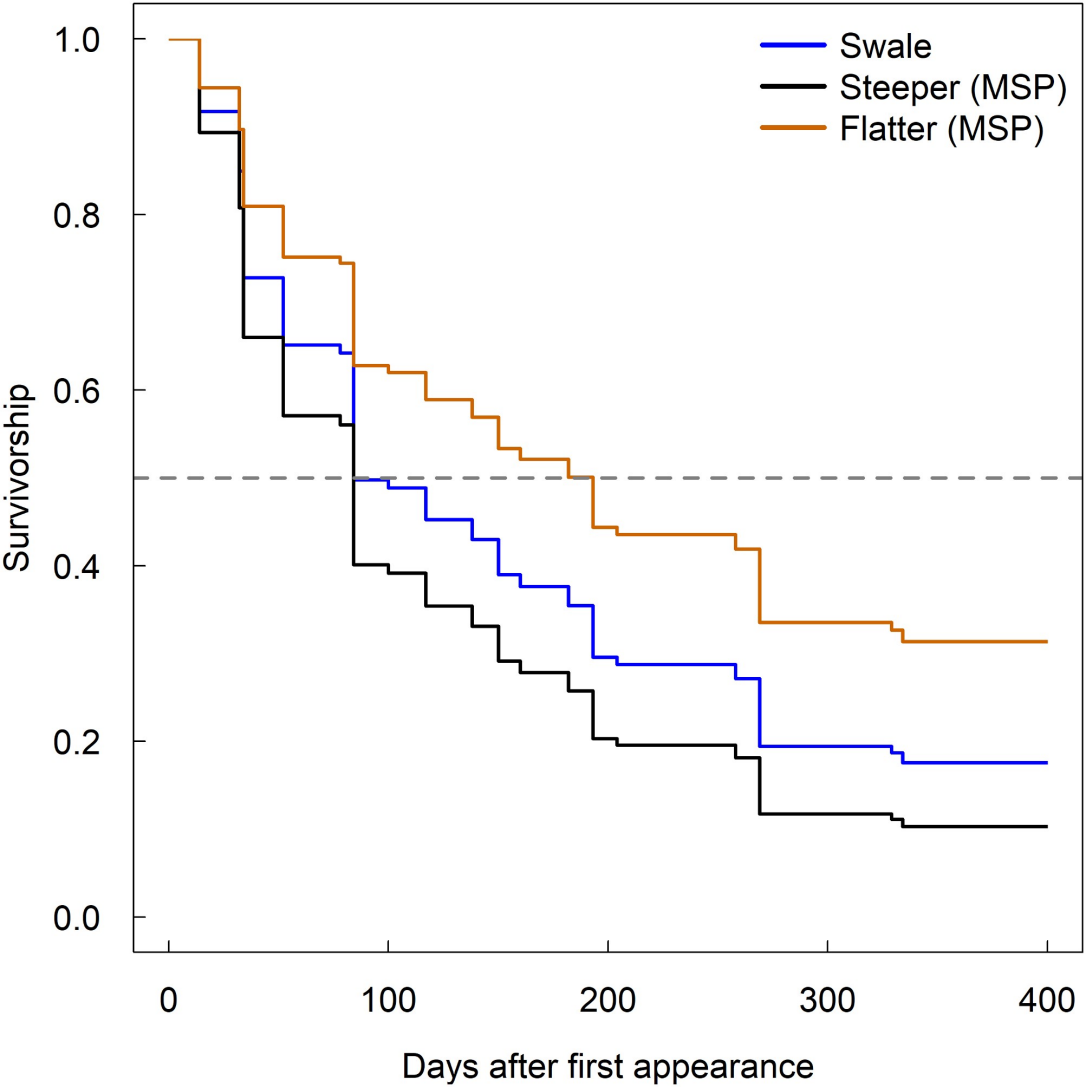
Influence of soil series on root survivorship in midslope locations. Survivorship curves of first-order roots in the top 10 cm of soil born in the spring of 2018 across soils on the north slope with different slope steepness, specifically: Steep Weikert (Steeper (MSP)), flatter Weikert (Flatter (MSP)), and Rushtown (Swales).

Across the midslope region of the catchment, differences in root lifespans were split between two groups of soil series. Wetter soils, Rushtown and flatter Weikert, had longer root lifespans than drier, steeper Weikert and Berks soils (Table 2 and Fig 4). There was largely only one soil type in the other topographic regions of the catchment.

**Table 2:** Mid-slope soil series proportional hazards table for different years and seasons.

Year	Season	Cohort (*n*)	Soil Series	Risk Ratio	Robust SE	P-value
2017	Spring	39	Flatter Weikert	0.86	0.12	0.20
2017	Spring	**187**	**Rushtown**	**0.59**	**0.18**	**< 0.01**
2017	Spring	188	Steeper Weikert	1.13	0.20	0.55
2017	Summer	**111**	**Flatter Weikert**	**0.67**	**0.16**	**0.01**
2017	Summer	**364**	**Rushtown**	**0.59**	**0.21**	**0.01**
2017	Summer	432	Steeper Weikert	1.31	0.20	0.18
2017	Fall	**122**	**Flatter Weikert**	**0.35**	**0.14**	**< 0.01**
2017	Fall	**276**	**Rushtown**	**0.37**	**0.17**	**< 0.01**
2017	Fall	331	Steeper Weikert	0.93	0.19	0.70
2018	Spring	**29**	**Flatter Weikert**	**0.42**	**0.26**	**< 0.01**
2018	Spring	**124**	**Rushtown**	**0.58**	**0.20**	**< 0.01**
2018	Spring	**98**	**Steeper Weikert**	**0.60**	**0.23**	**0.03**
2018	Summer	75	Flatter Weikert	0.66	0.22	0.06
2018	Summer	**318**	**Rushtown**	**0.45**	**0.17**	**< 0.01**
2018	Summer	453	Steeper Weikert	0.75	0.18	0.11
2018	Fall	**31**	**Flatter Weikert**	**0.50**	**0.25**	**< 0.01**
2018	Fall	339	Rushtown	1.08	0.29	0.79
2018	Fall	548	Steeper Weikert	1.09	0.26	0.75

Risk ratios of roots along the mid-slope. Different soil series were compared to the Berks soil series for roots born in spring, summer, and fall seasons in 2017 and 2018. Risk ratios > 1 shows that risk of mortality was increased at the location, suggesting that lifespan was shorter. Risk ratios < 1 shows that risk of mortality was decreased at the location. Bolded lines were statistically significant (*P*<0.05). Cohort sizes for Berks soil series in spring (56), summer (249) and fall (102) for 2017 and 46, 165, and 64 for 2018, respectively.

#### Slope aspect

**Fig 5:**
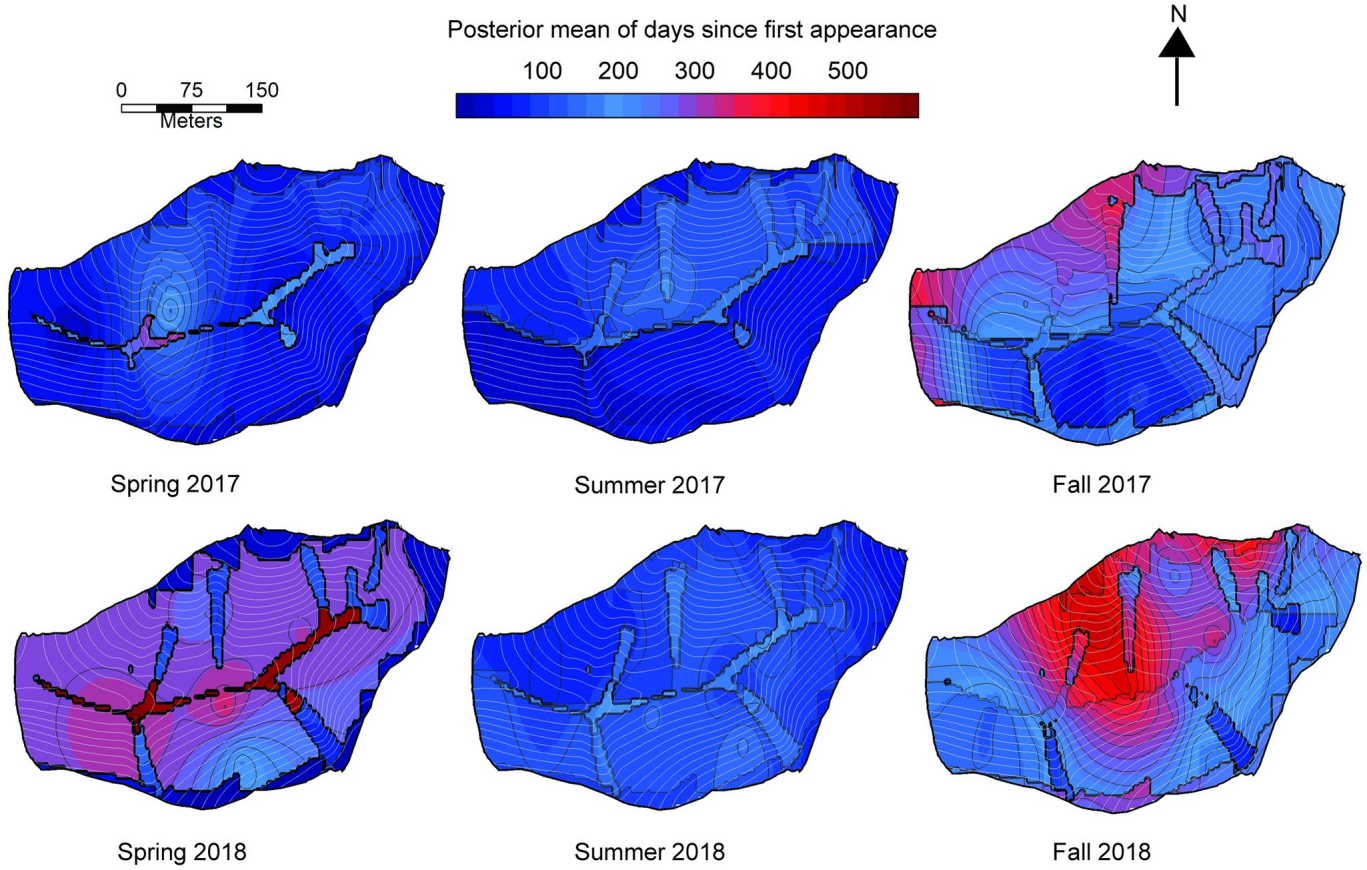
Seasonal trends in first-order root lifespan across the Susquehanna Shale Hills Critical Zone Observatory. Bayesian regression models were used in kriging. Maps displayed are posterior means of days since first appearance of a root in the upper 10 cm of soil. Number of plots with at least ten roots produced (*n*≥10) that were used in kriging were as follows: Spring 2017 (13 plots), summer 2017 (35), fall 2017 (25), spring 2018 (9), summer 2018 (38), and fall 2018 (27).

The influence of slope aspect on root survivorship shifted from year to year. In 2017, north-facing slopes had shorter root lifespans than south-facing slopes (Table 3). In 2018, there was generally no effect of aspect on root lifespan. However, for roots born in the spring of 2018, north-facing slopes had longer root lifespans than south-facing slopes. These trends were reflected in the regression kriging of fine root lifespan across the SSHCZO (Fig 5).

**Table 3:** Proportional hazards table for the effect of aspect (south or north facing) on root survivorship in different years and seasons.

Year	Season	Cohort of N-facing (*n)*	Cohort of S-facing (*n)*	Variable	Risk Ratio	Robust SE	P-value
2017	Spring	**222**	248	Aspect	**1.40**	**0.11**	**< 0.01**
2017	Summer	**507**	649	Aspect	**2.03**	**0.14**	**< 0.01**
2017	Fall	**369**	462	Aspect	**1.44**	**0.16**	**0.02**
2018	Spring	**138**	159	Aspect	**0.71**	**0.15**	**0.02**
2018	Summer	565	446	Aspect	1.03	0.14	0.81
2018	Fall	669	313	Aspect	1.47	0.27	0.16

Risk ratios for roots on the S-facing slopes when compared to N-facing slopes for roots born in the spring, summer, and fall seasons in 2017 and 2018. Risk ratios > 1 indicate that risk of mortality was greater at N-facing than S-facing slopes, suggesting that lifespan was shorter at the N-facing slope. Risk ratios < 1 show that risk of mortality was decreased at N-facing slopes compared to S-facing slopes. Bolded text was statistically significant (*P*<0.05).

#### Season

Summer-born roots had the shortest lifespans and had the highest associated risk ratios across years (Table 4). Spring-born roots had the next highest risk. Seasonal effects were shown across the SSHCZO via regression kriging of median lifespans of fine roots (Fig 5). The kriged map supported the Cox models in showing that fine root lifespan across the catchment tended to be shorter if the roots were born in the summer relative to other seasons.

**Table 4:** Season of birth proportional hazards table for different years.

Year	Cohort (*n)*	Season	Risk Ratio	Robust SE	P-value
2017	870	Spring	**1.32**	**0.12**	**0.02**
2017	1644	Summer	**1.61**	**0.09**	**< 0.01**
2018	481	Spring	1.15	0.18	0.43
2018	1554	Summer	**1.37**	**0.13**	**0.01**

Risk ratios for roots born in the spring and summer seasons relative to the fall season in 2017 and 2018. Risk ratios > 1 show that risk of mortality was increased at that time of year compared to the fall season of birth, suggesting that lifespan was shorter. Risk ratios < 1 show that risk of mortality was decreased at that season of birth. Bolded text was statistically significant (*P*<0.05). Cohort sizes for fall seasons in 2017 (1237) and 2018 (1442).

## Discussion

### Topography

Over 2017 and 2018, in 3 of 6 and 4 of 6 seasons, respectively, swale and valley floor locations had significantly longer root lifespans than the drier ridge top and midslope planar locations. This generally supported our hypothesis that wetter sites would have longer lifespans if the soils were not too wet. Swale and valley floor locations generally have greater average seasonal soil moisture content. They also tend to have deeper soils [23] and often had greater leaf litter accumulation [22] and consequentially, greater nutrient inputs. This makes these sites more favorable for plant growth not only because of greater average water content, but also other aspects that improve soil fertility. Despite differences in species distribution across the catchment [22], our analyses did not indicate that a functional tree group (i.e., trees with thin fine root diameters that associate with AM mycorrhizal fungi, etc.) exerted a strong influence on community estimates of root lifespan. Our findings differ from others suggesting a species or root diameter effect [32,36].

Contrary to our hypotheses, we did not find that excessively wet locations that might induce anoxic effects, such as deeper soil valley floor locations in the spring, resulted in shortened fine root lifespans. This is contrary to what others have shown [24,25], likely because while these areas did experience flooding events, flooding did not persist long and occurred when soil temperatures were low and when root metabolic activity is expected to be low. Certain minirhizotron tubes and sites experienced more saturated soil conditions that could be seen by the water level visible in the minirhizotron tube (personal observation). Even though in the spring there would often be tubes where a water level could be seen near the surface, roots below this level did not seem to suffer anoxic effects leading to their death. Instead, swale and valley floor sites supported longer fine root lifespans similar to other studies examining increased water effects on lifespan [28].

Our hypothesis that root lifespans would be impacted by soil water content, while supported in the topographic regions, was not supported when we tested for the effect of short term water content variation impacts on fine root lifespan. We found no effect of biweekly soil water content changes, regardless of magnitude, on fine root lifespan. This is contrary to what has been shown previously [28]. It could be that trees in swales and valley floor regions maintained roots in flooded soils by limiting their metabolism and relying on roots in drier soils during flooded periods. Ridge top locations, the driest sites, generally did not show significant differences in root lifespan from the midslope planar locations, suggesting that the long-term differences in soil moisture between the two topographic locations did not seriously impact root lifespan. Alternatively, tree root systems could have spread out wide enough from the ridgetop region into the midslope or swale regions that the drier soils along the ridgetop could be remediated via hydraulic redistribution [55]. Distances ranged from 5–10 meters at most between the ridgetop sites and the midslope planar or swale locations. Tree roots have been shown to be able to spread their roots up to 50 m away from their trunks and average around 8 meters away [56]. It was unclear what processes resulted in increased mortality risk on the ridgetops for roots born in the spring of 2018.

Our results suggest that landscape- and ecosystem-scale models utilizing aboveground attributes to estimate average annual fine root lifespan could yield incorrect estimates when averaging over complex topography. Others have suggested that prediction of belowground attributes by aboveground attributes is too simplified [57]. If one calculated the median annual fine root lifespan for the SSHCZO using this data set, based on a “representative” location such as the midslope planar location, the median lifespan would be 136 days in 2017 and 123 days in 2018. Using a weighted median of the SSHCZO based on estimated contributing area of contributing regions in the catchment, the median lifespan is closer to 201 days and 179 days in 2017 and 2018, respectively. This suggests that ignoring complex topography when estimating catchment-level fine root lifespan could result in estimates being off by roughly two months, or approximately 46%. This level of error is present prior to accounting for season of birth effects in addition to topographical variables. Errors in estimating fine root lifespan across landscapes could result in the misestimation of the impact of fine roots on soil C flux and ultimately, temperate forest ecosystem C flux. A caveat to this finding is that landscapes that consist of mainly one type of topographic variable, such as a landscape with mostly midslope planar type topography, then potentially one representative location could be acceptable. Due to the heterogeneity in root presence from tube to tube, we recommend never using one site for minirhizotron data when describing a hilly landscape. Moreover, our data suggest that models which are based on short-term changes in soil water content and temperature will not be particularly useful for estimating root lifespan.

### Depth

Across seasons and years, deeper roots consistently had longer root lifespans than shallow roots, as shown previously [29–32]. Increasing soil depth is correlated to many variables that have been shown to increase fine root lifespan, such as increased soil water content [28] and decreased temperature fluctuations [58]. The effect of depth on fine roots was such that fine root lifespan tripled from roughly a year to beyond three years when comparing roots living in upper centimeters of the soil to roughly a meter.

### Soil series

For topographic locations where there were more than one soil series present, we found soil classification type was useful in accounting for variation in fine root lifespan. Baldwin et al., [23] suggested that utilizing a combined soil-terrain-unit map would be better at predicting soil moisture content over time. Based on their map of this catchment, we classified our macroplot locations into their own soil series class based on a combination of slope class and soil series. Macroplot locations with flatter slopes and Weikert soils showed similar risk ratios to swale locations. Additionally, Weikert soils with steeper slopes had higher associated risk ratios and performed more similarly to Berks soils within the SSHCZO. Nonetheless, slope alone nor depth to tube refusal or maximum tube depth in the midslope region could account for significant variation in root lifespan. Perhaps these soil series classifications capture an unmeasured variable in addition to slope and soil depth that significantly contributed to the influence of soil series on root lifespan.

### Aspect

North-facing slopes had shorter root lifespans in 2017, but not in 2018, with the exclusion of spring 2018. Trees on the S-facing slope had access to three swales and gentler slopes along this face, which may have allowed trees to sustain their roots for a longer period of time than trees on the steeper, N-facing slopes. Wetter years may have diminished the influence of swale number and slope steepness leading to the observed lifespan differences observed in 2017 and 2018 lifespan. In 2017, the median root lifespan was 330 d for the S-facing slope and only 89 d for the N-facing slope, a considerable difference. Such differences in fine root lifespan on different aspects could substantially affect estimates of root lifespan in temperate forests with complex topography. No other studies to our knowledge have shown an effect of aspect on root lifespan.

### Season

We found that season had a strong impact on root lifespan, with roots born in fall having the longest lifespan and roots born in the summer having the shortest. Other studies have also shown that the season a root was produced impacted fine root lifespan [7,35,36,59]. The differences in fine root lifespan between summer and the other seasons could be related to increased photosynthesis rates and heat during the summer. More metabolically active roots or roots that are taking up nutrients at a faster rate tend to have shorter fine root lifespans [60]. Thus, roots born in the summer may need to be replaced more often than roots born in other seasons if they are more metabolically active. Conversely, some studies have shown that fine roots tend to have shorter lifespans when exposed to soil frost [61]. We generally did not see a substantial increase in the risk ratio for roots born in the fall relative to the other seasons, suggesting that in our system we did not have strong, soil-frost effects. This could be due to generally strong litter cover in the fall and usually a snow cover across the study area (personal observation).

### Soil water content and root related variables

#### Soil water content

We found our hypothesis that volumetric water content changes over time would have a consistent influence on root lifespan to be unsupported, in contrast to acute or short-term studies [28]. We found that wetter topographic regions tended to have longer fine root lifespans; however, this was unrelated to the short-term changes in the soil water content. This likely indicates that either tree root systems have spread enough to be able to support fine roots in dry conditions [55] or trees have altered the morphological and physiological traits of their roots to allow them to be acclimated to the range of water conditions present around them [62], or there are other, more limiting variables affecting root lifespan than soil water content conditions.

#### Root related variables

Number of root neighbors was not found to consistently influence root lifespan. Nor was fine root diameter found to significantly impact root lifespan. This contrasts with other studies looking at fine root diameter [31,32,59] and root neighbors [52,59]. This could be due to our observations being taken from a mixed forest compared to more controlled environments in previous studies. One group has suggested that thinner diameter roots may be able to modulate their lifespans under more optimal conditions [59], which may explain why we saw no effect of root diameter. Alternatively, we used root disappearance or visible root degradation as an indication of root mortality. It is possible that some of the fine roots at deeper depths and at different sites degraded at a slower rate relative to other locations, which would give the appearance of longer fine root lifespans, even though they may no longer be viable. Decomposition tends to be slower at dry sites [63], which would suggest that ridge top locations could have slightly shorter lifespans than reported here, which only accentuates our findings that the wetter locations had longer fine root lifespans.

## Conclusions

Root lifespans were substantially longer in topographic regions that tend to be wetter and have deeper soils like swales and valley floor. This could lead to erroneous catchment-scale estimates if, for example a planar hillslope was taken as a “representative” location. We also found strong depth, soil series, slope-facing aspect, and seasonal effects on root lifespan. Complex topography, which may include changing soil series type and slope aspect can shift root lifespan in temperate forest, depending on the year. Our work suggests that accounting for complex topography in models may improve prediction of root survivorship.

## Supporting information

S1 File Table of means and ranges for abiotic site variables, proportional hazards table for topography by depth interaction, and proportional hazards table for depth across years and seasons of birth. 1) Years, depths, means, standard errors, and ranges for environmental and root variables measured at the sites during the years of the study and prior. 2) Risk ratios for roots present at different topographic regions, depths, and topography by depth interactions, specifically depth × ridge top, depth × swale, and depth × valley floor. Depth × Midslope planar is the comparison. 3) Risk ratios for roots present at increasing depth ranks (0–10 cm, 10–25 cm, 26–34 cm, 34–40 cm, 40–60 cm, and > 60 cm) born during spring, summer, and fall of 2017 and 2018.
(DOCX)
