# Supplementary material for: Topographical shifts in fine root lifespan in a mixed, mesic temperate forest
Source: PLoS One. 2021 Jul 14;16(7):e0254672. doi: 10.1371/journal.pone.0254672 (PMC8279377; doi:10.1371/journal.pone.0254672)
Supplement: S1 File — 1) Years, depths, means, standard errors, and ranges for environmental and root variables measured at the sites during the years of the study and prior. 2) Risk ratios for roots present at different topographic regions, depths, and topography by depth interactions, specifically depth × ridge top, depth × swale, and depth × valley floor. Depth × Midslope planar is the comparison. 3) Risk ratios for roots present at increasing depth ranks (0–10 cm, 10–25 cm, 26–34 cm, 34–40 cm, 40–60 cm, and > 60 cm) born during spring, summer, and fall of 2017 and 2018. (DOCX) [file pone.0254672.s001.docx]

# **Supplemental**

**Table S1.**

| **Year** | **Variable** | **Mean** | **Standard Error** | **Range** |
| --- | --- | --- | --- | --- |
| 2014 | NO_3_  0-20 cm | 0.08 ppm | 0.01 | <0.01 – 0.72 ppm |
| 2014 | NO_3_  20-40 cm | 0.04 ppm | <0.01 | <0.01 – 0.20 ppm |
| 2014 | NO_3_  40-100 cm | 0.03 ppm | <0.01 | <0.01 – 0.12 ppm |
| 2014 | NH_4_ 0-20 cm | 1.24 ppm | 0.09 | 0.20 – 8.22 ppm |
| 2014 | NH_4_ 20-40 cm | 0.39 ppm | 0.02 | 0.16 – 1.34 ppm |
| 2014 | NH_4_ 40-100 cm | 0.26 ppm | 0.02 | 0.13 – 0.66 ppm |
| 2014 | Soil organic matter (SOM) 0-20 cm | 7.34% | 0.17 | 2.81 – 13.31% |
| 2014 | Soil organic matter (SOM) 20-40 cm | 3.82% | 0.05 | 2.89 – 5.51% |
| 2014 | Soil organic matter (SOM) 40- 100 cm | 3.34% | 0.06 | 2.69 – 4.68% |
| 2017 | Leaf litter | 34.52 g m^-2^ | 1.24 | 17.62 – 64.54 g m^-2^ |
| 2018 | Leaf litter | 109.69 g m^-2^ | 3.21 | 52.57 – 156.44 g m^-2^ |
| 2017-2018 | Tube depth | 57.4 in | 2.22 | 14.7 – 97.9 in |
| 2017 | Number of root neighbors | 6 | 0.04 | 0 - 51 |
| 2018 | Number of root neighbors | 7 | 0.07 | 0 - 51 |
| 2017 | Root diameter | 0.31 mm | <0.01 | 0.2 - 1.94 mm |
| 2018 | Root diameter | 0.30 mm | <0.01 | 0.2 – 1.96 mm |
| 2017 | Unscaled soil volumetric water content (uVWC) | 0.29 v/v | <0.01 | 0.11 - 0.55 v/v |
| 2018 | uVWC | 0.32 v/v | <0.01 | 0.04 – 0.55 v/v |

**Table S2.**

| **Year** | **Season of birth** | **Variable** | **Risk Ratio** | **Robust SE** | **P-value** |
| --- | --- | --- | --- | --- | --- |
| 2017 | Spring | Depth | **0.68** | **0.14** | **< 0.01** |
| 2017 | Spring | Ridge | 1.16 | 0.46 | 0.74 |
| 2017 | Spring | Swale | 0.87 | 0.31 | 0.66 |
| 2017 | Spring | Valley | 0.47 | 0.47 | 0.11 |
| 2017 | Spring | Depth × Ridge | 0.83 | 0.26 | 0.48 |
| 2017 | Spring | Depth × Swale | 0.84 | 0.16 | 0.28 |
| 2017 | Spring | Depth × Valley | 0.85 | 0.24 | 0.51 |
| 2017 | Summer | **Depth** | **0.75** | **0.10** | **<0.01** |
| 2017 | Summer | Ridge | 0.93 | 0.29 | 0.80 |
| 2017 | Summer | Swale | 0.69 | 0.25 | 0.14 |
| 2017 | Summer | Valley | 0.76 | 0.28 | 0.33 |
| 2017 | Summer | Depth × Ridge | 0.99 | 0.15 | 0.93 |
| 2017 | Summer | Depth × Swale | 0.96 | 0.13 | 0.78 |
| 2017 | Summer | **Depth × Valley** | **0.71** | **0.16** | **0.03** |
| 2017 | Fall | **Depth** | **0.61** | **0.12** | **<0.01** |
| 2017 | Fall | Ridge | 0.69 | 0.29 | 0.20 |
| 2017 | Fall | Swale | 0.55 | 0.32 | 0.06 |
| 2017 | Fall | Valley | 0.93 | 0.21 | 0.74 |
| 2017 | Fall | Depth × Ridge | 1.16 | 0.22 | 0.49 |
| 2017 | Fall | Depth × Swale | 1.08 | 0.15 | 0.61 |
| 2017 | Fall | Depth × Valley | 0.84 | 0.15 | 0.25 |
| 2018 | Spring | **Depth** | **0.60** | **0.19** | **0.01** |
| 2018 | Spring | Ridge | 2.01 | 0.49 | 0.15 |
| 2018 | Spring | Swale | 1.55 | 0.41 | 0.28 |
| 2018 | Spring | Valley | 0.71 | 0.53 | 0.52 |
| 2018 | Spring | Depth × Ridge | 0.96 | 0.28 | 0.88 |
| 2018 | Spring | Depth × Swale | 0.74 | 0.22 | 0.17 |
| 2018 | Spring | Depth × Valley | 0.68 | 0.31 | 0.23 |
| 2018 | Summer | **Depth** | **0.73** | **0.12** | **0.01** |
| 2018 | Summer | Ridge | 1.28 | 0.84 | 0.77 |
| 2018 | Summer | Swale | 0.68 | 0.42 | 0.35 |
| 2018 | Summer | Valley | 1.06 | 0.41 | 0.89 |
| 2018 | Summer | Depth × Ridge | 0.49 | 0.66 | 0.28 |
| 2018 | Summer | Depth × Swale | 0.97 | 0.22 | 0.89 |
| 2018 | Summer | Depth × Valley | 0.63 | 0.26 | 0.08 |
| 2018 | Fall | Depth | 0.83 | 0.16 | 0.26 |
| 2018 | Fall | Ridge | 4.34 | 0.82 | 0.07 |
| 2018 | Fall | Swale | 0.96 | 0.40 | 0.92 |
| 2018 | Fall | Valley | 0.79 | 0.43 | 0.58 |
| 2018 | Fall | **Depth × Ridge** | **0.21** | **0.54** | **<0.01** |
| 2018 | Fall | Depth × Swale | 1.05 | 0.19 | 0.80 |
| 2018 | Fall | Depth × Valley | 0.90 | 0.34 | 0.75 |

Risk ratios > 1 shows that risk of mortality was increased at the location, suggesting that lifespan was shorter. Risk ratios < 1 shows that risk of mortality was decreased at the location. Bolded lines were statistically significant (*P*<0.05).

**Table S3.**

| **Year** | **Season of birth** | **Variable** | **Risk Ratio** | **Robust SE** | **P-value** |
| --- | --- | --- | --- | --- | --- |
| 2017 | Spring | Depth | **0.60** | **0.08** | **< 0.01** |
| 2017 | Summer | Depth | **0.71** | **0.06** | **< 0.01** |
| 2017 | Fall | Depth | **0.61** | **0.07** | **< 0.01** |
| 2018 | Spring | Depth | **0.49** | **0.09** | **< 0.01** |
| 2018 | Summer | Depth | **0.66** | **0.14** | **< 0.01** |
| 2018 | Fall | Depth | **0.78** | **0.10** | **0.02** |

Risk ratios > 1 shows that risk of mortality was increased at the location, suggesting that lifespan was shorter. Risk ratios < 1 shows that risk of mortality was decreased at the location. Bolded lines were statistically significant (*P*<0.05).
